# Supplementary material for: Compact terahertz harmonic generation in the Reststrahlenband using a graphene-embedded metallic split ring resonator array
Source: Nat Commun. 2024 Mar 14;15:2312. doi: 10.1038/s41467-024-45267-2 (PMC10940712; doi:10.1038/s41467-024-45267-2)
Supplement: Supplementary file 1 — Supplementary Information [file 41467_2024_45267_MOESM1_ESM.pdf]

Supplementary Information

Compact terahertz harmonic generation in the Reststrahlenband using a graphene-embedded metallic split ring resonator array

Alessandra Di Gaspare[1], Chao Song,[2] Chiara Schiattarella,[1] Lianhe H. Li[3], Mohammed Salih[3] A. Giles Davies,[3] Edmund H. Linfield[3], Jincan Zhang[4], Osman Balci[4], Andrea C. Ferrari[4], Sukhdeep Dhillon[2] and Miriam S. Vitiello[1]

[1]NEST, CNR-NANO and Scuola Normale Superiore, 56127, Pisa, Italy
[2]Laboratoire de Physique de l'Ecole Normale Supérieure, ENS, Université PSL, CNRS, Sorbonne Université, Université de Paris, Paris, France
[3]School of Electronic and Electrical Engineering, University of Leeds, Leeds, LS2 9JT, UK
[4]Cambridge Graphene Centre, University of Cambridge, Cambridge, CB3 0FA, UK

Supplementary Note 1. Optimization of optical band of CSRR

The CSRR design is optimized as follows. Initially, a split ring is engineered, varying the ring geometric parameters (external and internal radii). We then select 3 different geometries (Supplementary Fig.1), having resonances at 2.8, 3, 3.2 THz, and fabricate 3 samples based on the corresponding designs, with SLG coating over the whole SRR area.

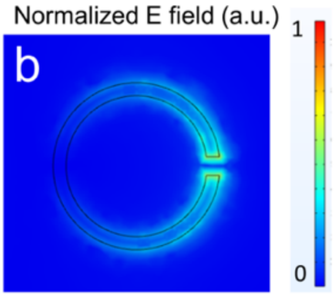

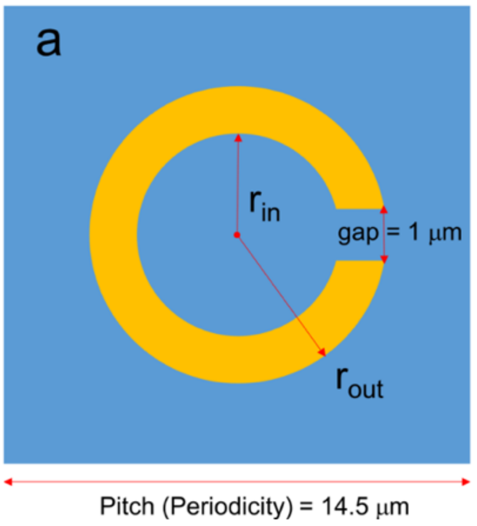

c

r_{in} (μm)	r_{out} (μm)	ν_0 (THz)
3.8	4.5	2.8
3.6	4.2	3.0
3.1	4.1	3.2

Supplementary figure 1. Circular Split-Ring Resonator design. a) Sketch of the unit cell of the CSRR design used in the finite element method (FEM) simulations, view from the top. The yellow region is modeled as a perfect electric conductor, while the blue area is the dielectric substrate. b) Color map of the absolute electric field distribution extracted by the simulation of the resonator in a), at ν_0=3.0 THz. c) Geometrical parameters of the CSRR, i.e. internal and external radii and the corresponding resonance frequencies extracted from the FEM simulation of the ring in a), maintaining a split gap size of 1μm.

Supplementary figure 2 plots the FTIR transmittance from each sample, with and without SLG in the gap, revealing a bleaching in the SLG-coated sample, attributed to the resonator screening from the semi-metallic SLG covering the entire surface.

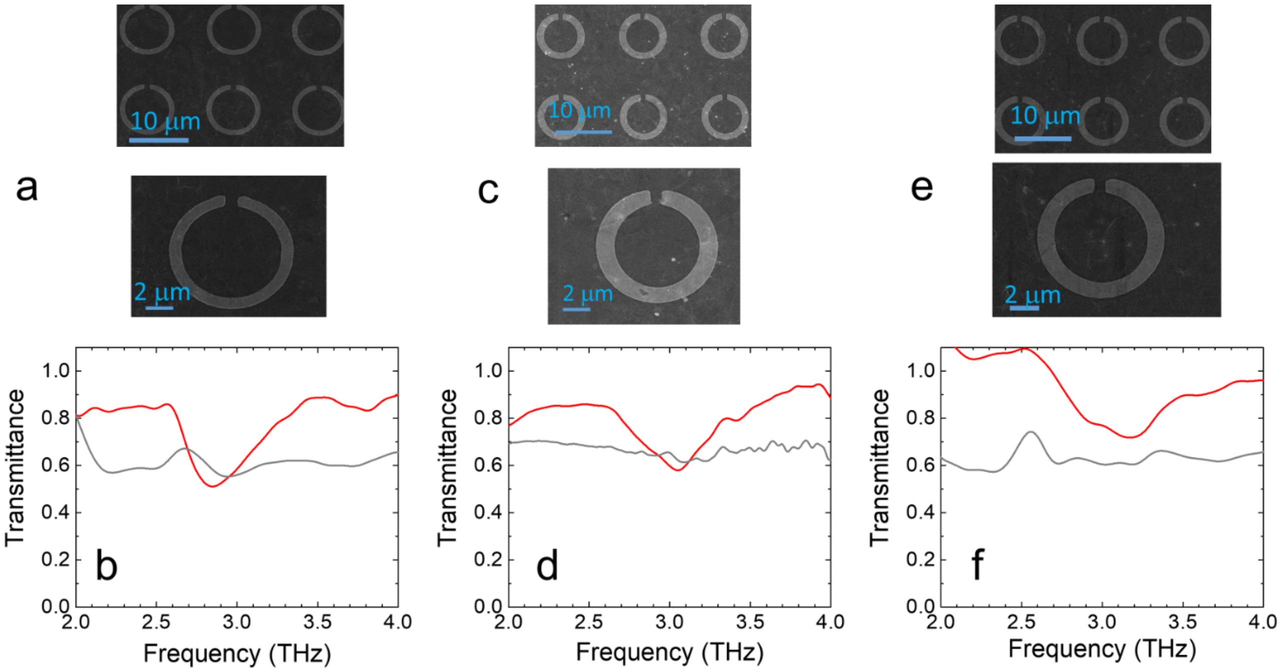

Supplementary figure 2. CSRR arrays transmittance: Scanning electron microscope images (top panels) and normalized transmittance measured via FTIR spectroscopy (bottom panels) of CSRR array of Supplementary Fig.1a, fabricated with the geometrical parameters of Supplementary Fig.1c, showing resonances at (a,b) ν_0=2.8 THz, (c,d) 3.0 THz, (e,f) 3.2 THz. The FTIR transmittances are acquired on both uncoated CSRR (red curves) and SLG-CSSR (gray curves), with SLG coating the entire CSRR array surface.

Supplementary Note 2. Raman spectroscopy

Raman spectroscopy is used to monitor the SLG quality and doping.[1-3] Raman spectra are acquired with a Renishaw Invia on as-grown SLG/Cu at a 514 nm using a 100 × objective with an optical power density <0.5 mW/μm^2 to prevent sample heating, Supplementary Fig. 3a. We get Pos(G) =1586 ± 4 cm^{-1} and Pos(2D) = 2698 ± 8 cm^{-1} from a statistical analysis of 5 spectra at different points on the sample surface. The 2D and G peaks are single Lorentzians with full-width-at-half-maximum FWHM(2D) = 49 ± 10 cm^{-1}, and FWHM(G) = 19 ± 3 cm^{-1}. The 2D to G peak intensity and area ratios are I(2D)/I(G) = 1.85 ± 0.3 and A(2D)/A(G) = 4.64 ± 0.8, corresponding to p doping~300meV.[4] I(D)/I(G) = 0.07 ± 0.07. Raman spectra are then measured on SLG on the CSRR, Supplementary Fig. 3b. We get Pos(G)=1594 ± 6 cm^{-1}, POS(2D)=2675 ± 4 cm^{-1}, from a statistical analysis on 5 different points on the sample surface. A D peak is also visible, with I(D)/I(G) = 0.92 ± 0.02, indicating the presence of Raman active defects, possibly associated with the processing.[1-3] FWHM(G) = 18 ± 1 cm^{-1}, I(2D)/I(G) = 1.98 ± 0.7, A(2D)/A(G) = 3.8 ± 0.7, corresponding to E_F = 250 ± 70 meV[4,5].

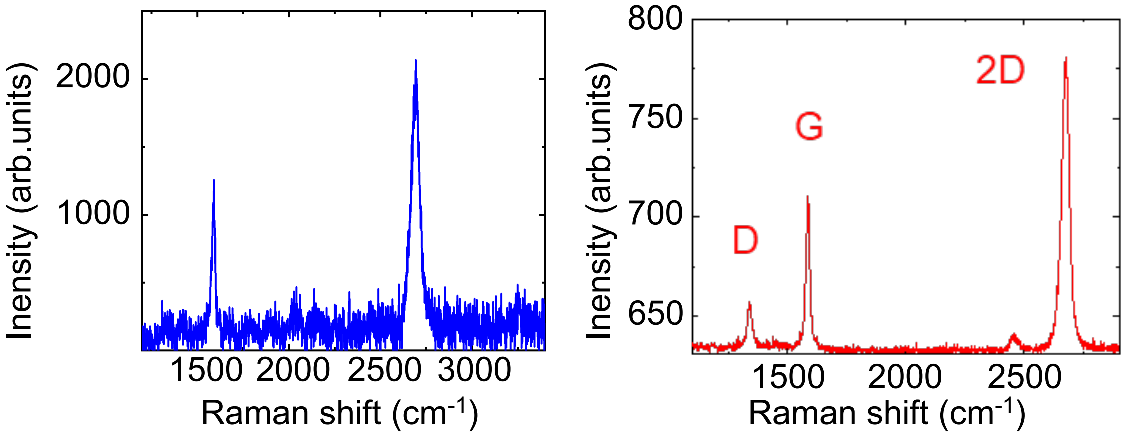

Supplementary figure 3. Single Layer Graphene Raman analysis: 514.5nm Raman spectra of a) SLG on Cu at 514 nm, b) SLG in CSRR array.

The carrier mobility value was extracted from IV transport curves carried out on SLG Field Effect Transistors, realized with SLG from the same CVD batch, processed simultaneously to the SRR sample preparation, and using a SiO_2 substrate, as for the SRR sample[7]. Both doping level extracted by micro-Raman spectra and the carrier mobility are in agreement with the typical values retrieved on large area, polycrystalline SLG transferred on dielectric substrates.

Supplementary Note 3. Simulation with SLG in the gap region

The total SLG optical conductivity $\sigma_{SLG}(\omega)$ is the sum of intraband and interband terms[7]: $\sigma_{SLG}(\omega) = \sigma_{intra}(\omega) + \sigma_{inter}(\omega)$. In the THz region, the optical response is dominated by intraband absorption, and we calculate the SLG conductivity according to the Drude model:[8,9]

$$\sigma_{Drude}(\omega) = \sigma_{DC}\frac{1}{1-i\omega\tau} \tag{1}$$

where τ is the scattering time, $\sigma_{DC} = \frac{2e^2}{h}|k_F|v_F\tau$ is the static conductivity of Dirac fermions in graphene, $v_F = 1 \times 10^6$ m/s is the Fermi velocity, k_F is the Fermi momentum, defined as $k_F = \sqrt{\pi n}$, and n is the 2d electron gas density of the Dirac system. τ is related to the mobility μ through the relation: $\tau = \frac{\mu E_F}{e v_F}$. The three-dimensional conductivity in SLG can be written as[6] $\sigma_{SLG} = \frac{\sigma_{Drude}(\omega)}{d}$, where d=0.335 nm is the SLG thickness. The complex dielectric function for SLG on a dielectric substrate with refractive index n_{sub} is[10]:

$$\varepsilon_{SLG}(\omega) = 1 + \frac{i\sigma_{SLG}(\omega)}{(n_{sub}+1)\varepsilon_0\omega} \tag{2}$$

The real and imaginary part of the complex refractive index are then defined as:

$$n_{SLG} = Re(\sqrt{\varepsilon_{SLG}(\omega)}) \tag{3}$$

$$k_{SLG} = Im(\sqrt{\varepsilon_{SLG}(\omega)}) \quad (4)$$

In our simulations SLG is treated as a transition boundary condition with complex refractive index from Eqs. 3,4, calculated using: n_{sub} =3.425, E_F=250 meV, μ=1100 cm^2/Vs, leading to τ=23.5 fs and σ_{DC}=0.43 mS, as SLG contribution to the total conductivity. The resulting n_{SLG}, k_{SLG} are in Supplementary Fig. 4.

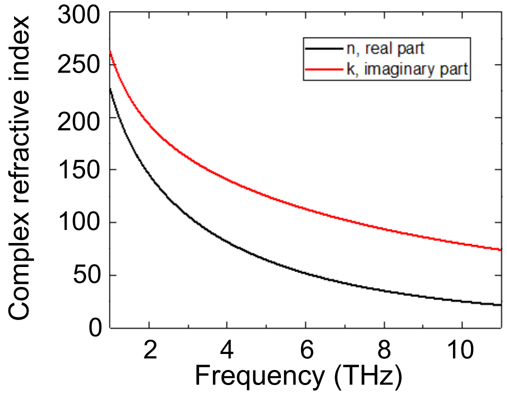

Supplementary figure 4. SLG optical constants: Real (black) and imaginary (red) parts of SLG with E_F=250 meV and τ=23.5 fs.

Supplementary Note 4. CSRR simulations

4.1 Extraction of core parameters

Finite element method simulations are done by implementing two ports, one transmitting and the other receiving the plane wave impinging on the CSRR array unit cell.

The frequency dependent transmittance is then extracted by the modulus squared of the S_{12} parameter, included in the simulation output functions. The central frequency ν_0 and the quality factor Q are then extracted through a Gaussian fit. Supplementary Fig.5a shows the transmittance, plotted as a function of frequency, calculated for the CSRR defined in Supplementary Fig.1a, while varying the gap size. The field enhancement factor is defined as the ratio between the optical field inside and outside the enhancement region. It is extracted by defining a 5 × 5 μm^2 domain across the gap region (see Supplementary Fig.6b), and then calculating the average value of the total field magnitude. A second 2d domain, having the same size, is defined at the center of the SRR, where there is no field enhancement, and used to extract the zero-enhancement average field (see Supplementary Fig.5c).

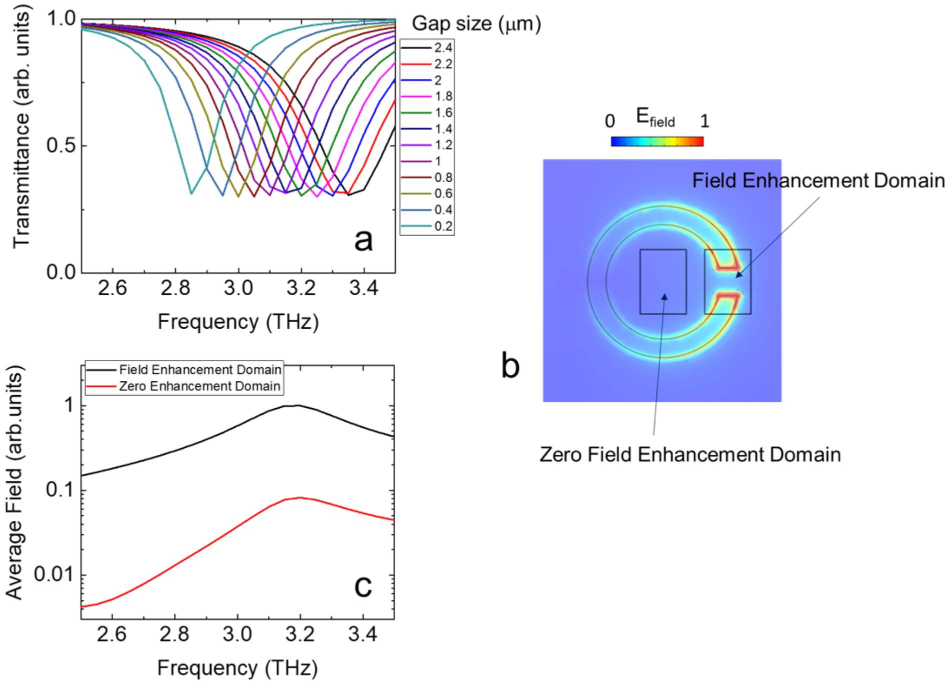

Supplementary figure 5. CSRR Resonance optimization a) Normalized transmittance of CSRR design in Fig.1a, calculated by varying the split gap size from 0.2 to 2.4 µm (step = 0.2 µm), and keeping the other geometrical features constant (internal and external radii, and unit cell size). **b)** Color map of normalized absolute electric field distribution extracted by the simulation of the resonator in a), at resonance. The two rectangular boxes highlight the regions chosen to extract the total field, calculated as the surface average of the absolute electric field: the central region is the zero-enhancement domain, while the box on the right is the enhancement domain. **c)** Surface average electric field, as a function of eigenmode frequency in the zero enhancement (red) and field enhancement (black) domains in b.

4.2 CSRR Q-factors

The CSRR Q factor depends on the inter-lattice distance, i.e. p. When the ring-ring distance is comparable or lower than the effective wavelength, $\lambda_{eff} = \lambda_0/n_{sub}$, where λ_0=107 µm is the radiation wavelength in vacuum and n_{sub} = 3.5 is the effective refractive index of the dielectric hosting the CSRR array, inter-ring cross talk may introduce radiative losses with a resulting resonance broadening[11,12].

We perform a set of simulations on the CSRR of Fig. 1b (main text), by sweeping the unit cell size from 10 to 45 µm, i.e. below and above $\lambda_{eff}\sim$ 30 µm. We then calculate the corresponding transmittance curves, Q, and field enhancement factors (Supplementary Fig.6). By suppressing the lattice radiative losses, Q increases, and the field enhancement factor is marginally affected.

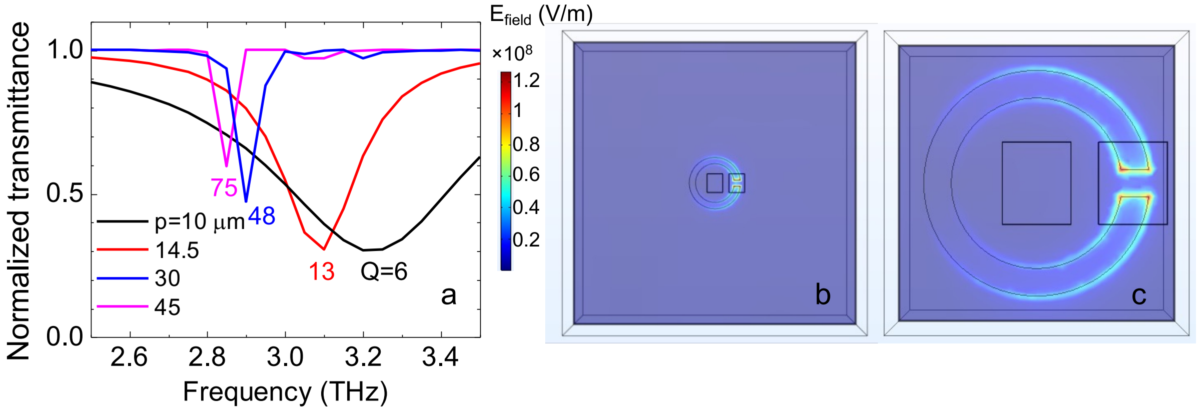

Supplementary figure 6. CSRR array Q-factors a) Transmittance spectra calculated by varying *p*: 10 μm (black), 14.5 μm (fabricated sample, red), 30 μm (blue) and 45 μm (magenta). b,c) Color map of total electric field distribution simulated on the SRR unit cell at resonance for the two extreme values of *p:* (b) 10 μm, (c) 45 μm.

4.3 High harmonic resonator modes

The optical response of the CSRR array considered here is expected to reflect the presence of both the fundamental resonance set by the design in Supplementary Fig.1, and higher order modes. The latter are expected to affect the THGE and must be taken into account. The transmittance calculated in the 3.6–12 THz range for the SRR design of Fig.1b of the main text has a resonance at the third harmonic (TH) frequency (see Fig.1d in the main text). The electric field distribution map at the TH frequency (Supplementary Fig.7a) reveals the multi-node character of this mode, still showing a significant field enhancement in the gap area, alongside two other hot spots on the ring. The calculated transmittance (Supplementary Fig.7b) reveals two sharp resonances in the range of interest for THG.

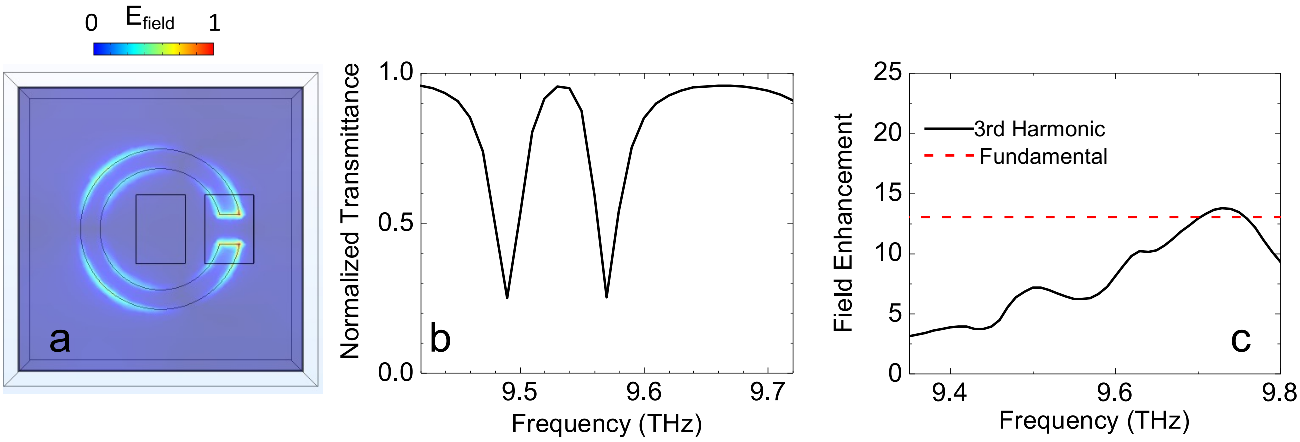

Supplementary figure 7. CSRR higher order modes a) Color map of normalized electric field at the higher order resonance of the CSRR transmittance in a) at ~9.6 THz. **b)** Transmittance calculated via high-frequency resolution simulation in the 9.4–9.8 THz range for the CSRR of a–b). **c)** Field enhancement for the 3rd harmonic mode of the CSRR (black). The curve is extracted through adjacent-averaging smoothing. The red dashed line highlights the enhancement factor for the fundamental mode.

The field enhancement factor, calculated with the same method, shows a value comparable with the fundamental mode (Supplementary Fig.7c). To quantify the effective field enhancement, we carry out the same Q analysis of Note 2.2, by performing FEM of the same CSRR with different p. We extract Q, Q_3, A_{G3}, as shown in Fig.3b.

Supplementary Note 5. Hot electron conductivity for 3rd order nonlinear response in SLG

5.1 Hot electron steady excitation state

The non-linear interaction between SLG free carriers and an intense driving THz electromagnetic field is described by the heating/cooling of the free carriers.[13,14] In the THz range, the intraband absorption in SLG of a high-power optical beam leads to a non-equilibrium state with an excess distribution of carriers at the energy of the optical pump. The ultrafast (~20 fs) carrier-carrier scattering[15] drives the initial energy redistribution, bringing the system into a non-equilibrium state with electrons sharing an hot-electron temperature T_e (Refs. 13,14). Then, the system relaxes back to an equilibrium state.[15] When the optical excitation (~µs in the present case) varies on a timescale much longer than the internal relaxation time of the SLG carriers (~ps)[16], a steady excitation state at T_e is realized, where:

$$T_e = T_{sub} + \frac{P_{in}\tau_{cool}}{C_e} \tag{5}$$

with T_{sub} is the temperature at equilibrium, P_{in} is the excitation power intensity, in units of Wcm^{-2}, and τ_{cool} is the cooling time. C_e, the SLG heat capacitance, can be expressed as:[13,14,17,18]

$$C_{e,doped} = \frac{2\pi E_F}{3(\hbar v_F)^2} k_B^2 T_e \tag{6}$$

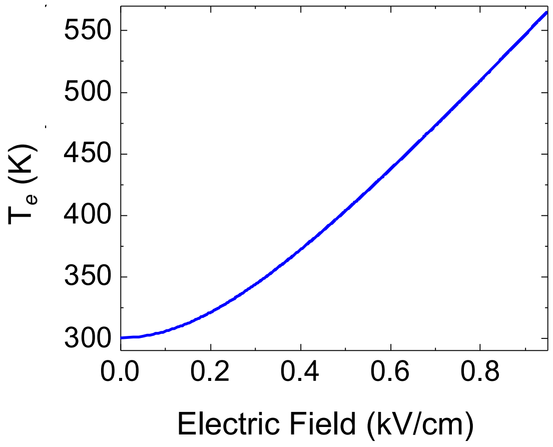

Supplementary figure 8. Hot-Electron Temperature in optically pumped graphene. Calculated hot electron temperature as a function of electric field according to Eqs. 5,6.

From previous reports on SLG on quartz, we assume τ_{cool}=2.5 ps[19,20,21], and by combining Eqs. 5 and 6, we extract the T_e dependence on the electric field (Supplementary Fig.8) at E_F =250 meV, as estimated by Raman spectroscopy.

5.2 Field-dependent conductivity

In presence of an intense excitation beam, the SLG nonlinear response can be expressed through a field-dependent conductivity:[22]

$$\sigma_{tot}(\nu) = \sigma_{intra}(\nu) + |E_0(\nu)|^2 \times \sigma_3(\nu) \tag{7}$$

where E_0 is the field intensity. The nonlinear term of the conductivity, σ_3, is the sum of the 3rd harmonic and Kerr effect terms:[22]

$$\sigma_3(\nu) = \eta[\sigma_{Kerr}(\nu)] \tag{8}$$

whose numerical expressions are:

$$\sigma_{Kerr}(\nu = \nu_{eff}) = \frac{i9e^6 v_F}{4\pi\hbar^4}\frac{D_{he}}{(2\pi\nu + i\Gamma_{he})(-2\pi\nu + i\Gamma_{he})(4\pi\nu + i\Gamma_{he})} \tag{9}$$

where the substitution of ν with $\nu_{eff} = (\nu^2 - \nu_0^2)/\nu$ accounts for the plasmonic LC resonance of the SRR, and η~0.6 is the total filling factor of the SLG-CSRR array. The E_F-dependent parameters D_{he} and Γ_{he} are the hot electron Drude weight and scattering rate, can be written as:[22]

$$D_{he} = D_0\left[1 - \frac{1}{6}\left(\frac{\pi k_B}{E_F}\right)^2 T_e{}^2\right] \tag{10}$$

$$\Gamma_{he} = \Gamma_0\left[1 + \frac{1}{6}\left(\frac{\pi k_B}{E_F}\right)^2 T_e{}^2\right] \tag{11}$$

The hot electron optical response is then captured by the transmittance coefficient, as for Eq.3 of the main text.

We use the Kerr effect to calculate the field-dependent, higher order terms, in the optical conductivity. This is the main factor for the observation of non-linear effects, depending on the strength of the driving electric field, and is at the origin of the observed THG signal. Furthermore, in eq. 7, the T_e-dependence was also considered for the intraband term, namely:

$$\sigma_{intra}(\nu) = \frac{-iD_{he}}{\pi}\frac{1}{(2\pi\nu + i\Gamma_{he})} \tag{12}$$

A quantitative comparison of the different weight of the Drude-like and field-driven terms in building up the graphene nonlinear response has been presented in our recent paper[23]. In Ref. 23 it is shown that in the un-patterned film (i.e. with no CSRR), the Drude thermal effect dominated over field-driven effects for moderate input power density/doping levels. Conversely, when accounting for the field enhancement of the CSRR, the field-driven third-order nonlinearity becomes the dominating term in establishing the THG efficiency, as visible from Supplementary Fig.9, showing the

comparison between the THG efficiency, calculated considering only the Kerr 3rd order term (blue curve), only the Drude hot-electron term (black curve), or the combination of both terms (red trace).

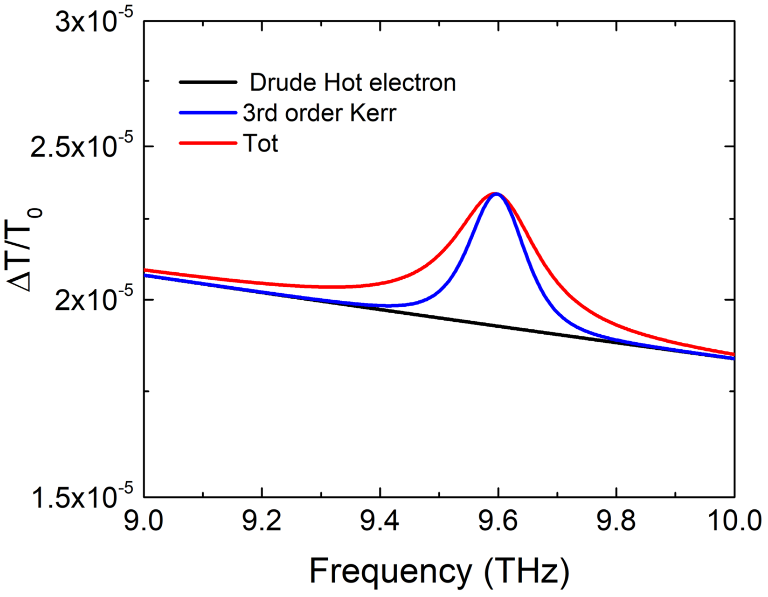

Supplementary figure 9. Third Harmonic Efficiency. $\Delta T/T_0$ ($3\nu_0$), i.e. THG efficiency, as a function of frequency in the pumped SLG-CSRR, calculated considering the 3rd order Kerr term (blue line), the hot-electron Drude like response (black line), or the combination of both terms (red trace).

5.3 Electric field in the SLG-CSRR array

The frequency dependent $E_0(\nu)$ in Eq.2 of the main text for the SLG-CSRR is calculated as follows. The external pump field, $E_{ext}(\nu)$ is a quasi-monochromatic, linearly polarized, radiation field at normal incidence, with P_{ext}=1 W focused on a d=700 μm diameter circular spot centered at ν_0 (see Inset in Fig.3c). We express such quantity as a Lorentzian peak normalized by the incoming power density in the focal region[22]

$$|E_{ext}(\nu)|^2 = \frac{Z_0 P_{ext}}{\left(\pi \frac{d^2}{4}\right)} \frac{l_w^2}{(\nu-\nu_0)^2+l_w^2} \tag{13}$$

where Z_0=377 Ω is the vacuum impedance, ν_0=3.2 THz is the external pump frequency and l_w=0.35 THz is its optical bandwidth. We calculate the total field enhancement in the SLG-CSRR as a function of frequency, $FE_{SRR}(\nu)$, as[24]:

$$FE_{SRR}(\nu) = A_G \times A_{SRR}(Q_0, Q_3, \nu) \tag{14}$$

where A_G is the average field enhancement factor of each SRR having different Q_0,Q_3 (see Fig.3b of main text), $A_G = [A_G(Q_0) + A_G(Q_3)]/2$. $A_{SRR}(Q_0, Q_3, \nu)$ is the normalized spectral absorption of the CSRR, including both fundamental and TH terms. By following the approach of Supplementary

Refs.23,24, we model the CSRR as a damped oscillator, whose optical response, owing to its magnetic character, is ascribed to a Lorentz dielectric function, i.e. a Lorentzian line-shape with Q defined by the resonator properties[25,26]:

$$\mu_{SRR}(\omega) = \mu_r \times \left(1 - \frac{{\omega_{pl}}^2}{\omega^2 - \omega_0^2 + i\Gamma_{SRR}\omega}\right) \quad (15)$$

where $(\omega; \omega_0) = 2\pi(\nu; \nu_0)$, $\omega_{pl} \sim 2\omega_0$ is the SRR plasma frequency[24], $\mu_r = 1$ is the out-of-resonance relative permeability and Γ_{SRR} is the resonator linewidth, set by the corresponding Q. The fundamental and TH resonances have the following dielectric functions[26,25]:

$$\mu_{SRR,0}(\omega) = \mu_r \times \left(1 - \frac{{\omega_{pl}}^2}{\omega^2 - \omega_0^2 + i\Gamma_{SRR,0}\omega}\right) \quad (16)$$

for the fundamental mode, and:

$$\mu_{SRR,3}(\omega) = \mu_r \times \left(1 - \frac{{\omega_{pl}}^2}{\omega^2 - (3\omega_0)^2 + i\Gamma_{SRR,3}\omega}\right) \quad (17)$$

for the TH resonance with linewidth $\Gamma_{SRR,3}$.

We use the CSRR Q at the fundamental (Q_0) and TH (Q_3) frequencies, as from the simulation results of Note 2.2, ranging from ~6 to 75 for Q_0 and from ~17 to 80 for Q_3. The absorption coefficient is calculated as[22]:

$$A_{SRR}(\omega) = \left| N \frac{\sqrt{\varepsilon_{sub}} - n_{SRR}}{\sqrt{\varepsilon_{sub}} + n_{SRR}} \right|^2 \quad (18)$$

where N is the normalization factor to set the $A_{SRR}(\omega)$ spectral function maximum ≡1, and n_{SRR} is the SRR complex refractive index, defined as $n_{SRR} = \sqrt{\varepsilon_{sub}\mu_{tot}(\omega)}$, where $\varepsilon_{sub} \approx 1.5$ is the average out-of-resonance dielectric constant of the substrate defined as $\varepsilon_{sub} = \left(\frac{n_{sub}+1}{2}\right)^2$. $\mu_{tot}(\omega) = [\mu_{SRR,0}(\omega) + \mu_{SRR,3}(\omega)]/2$ is the SRR effective dielectric function, including both fundamental and TH contributions. To extract $E_0(\nu)$, we multiply the electric field of Eq.12, $E_{ext}(\nu)$, by the frequency-dependent field enhancement of Eq.13, $FE_{SRR}(\nu)$:

$$|E_0(\nu)|^2 = |E_{ext}(\nu)|^2 \times |FE_{SRR}(\nu)|^2 \quad (19)$$

Supplementary Note 6. SLG-CSRR transmittance under optical pumping

Supplementary figure 10 compares the transmittances measured on the SLG-CSRR array without and with optical pumping. The SLG-CSRR array is mounted in the internal compartment of the FTIR, probed with the internal source of an FTIR spectrometer (Globar), under vacuum, in rapid scan mode (spectral resolution 1 cm^{-1}), without and with the QCL pump beam focused on the CSRR array with the help of an off axis parabolic mirror (focal length 15 cm), collecting the beam form the QCL facet, and a TPX lens (focal length 20 cm), focusing the beam to the sample surface on a spot of~0.7 mm diameter.

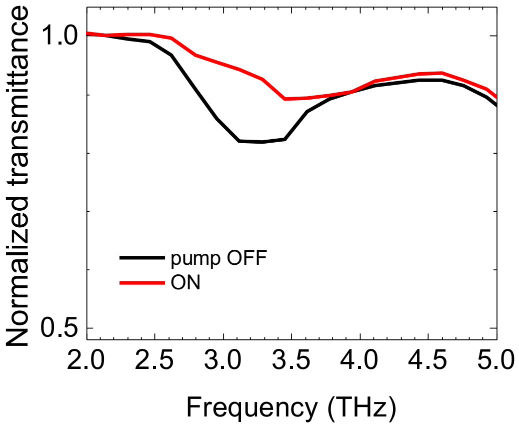

Supplementary figure 10. Optically Pumped SLG Transmittance. SLG-CSRR transmittance as a function of frequency, acquired by FTIR under vacuum with a spectral resolution of 1cm^{-1}, without (black) and with (red) QCL pump. The transmittances are normalized by the transmission traces acquired on a reference sample, i.e. the SiO_2/Si substrate used to fabricate the CSRR.

To avoid detecting the QCL signal directly on the detector, we tune the incidence angle so that the pump beam, after SRR surface reflection, does not travel along the interferometric path of the probe beam.

Supplementary Note 7. High-pass filter spectrum

Supplementary figure 11 is the normalized transmittance of the 2-mm-thick Ta high-pass filter (Crystan limited) used to isolate the THG term in the emission experiment (Fig.4 of main text). The cutoff frequency (υ_{co}) is defined as the frequency at which the transmittance is $T(\upsilon_{co}) \cong 0.7 \times T_{max}$, with υ_{co}=7 THz.

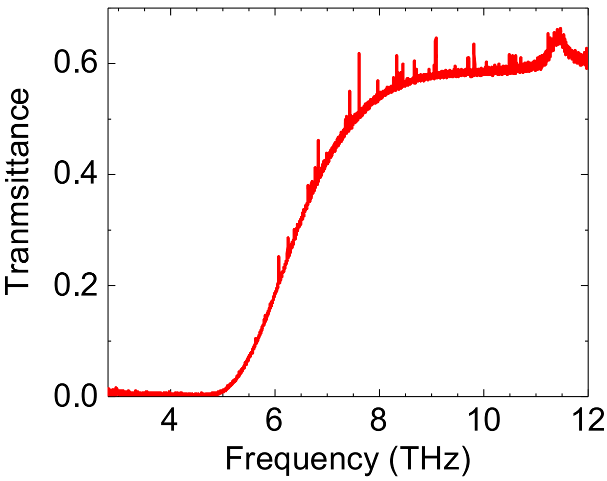

Supplementary figure 11. Ta-filter Transmittance. Transmittance of 2-mm-thick Ta high-pass filter employed for the suppression of the QCL fundamental mode acquired with a spectral resolution of 0.15 cm^{-1}. The trace is obtained by normalizing the curve acquired in transmission mode on the filter, with the internal source of the FTIR spectrometer as a reference, under vacuum.

Supplementary Note 8. Electro-Magnetic Coupling in CSRR

The electromagnetic response of the CSRR is dominated by the magnetic coupling, resulting from the induction current in the circular ring geometry. However, the magnetic coupling enhancement region of the CSRR design is spatially separated from the electric field area, as shown in Figs. 12 plotting the bi-dimensional distribution, at resonance, of the electric (Supplementary Fig. 12a) and magnetic fields (Supplementary Fig. 12b). Embedding graphene in the split gap area, allows exploiting the electric field enhancement to drive an efficient THG.

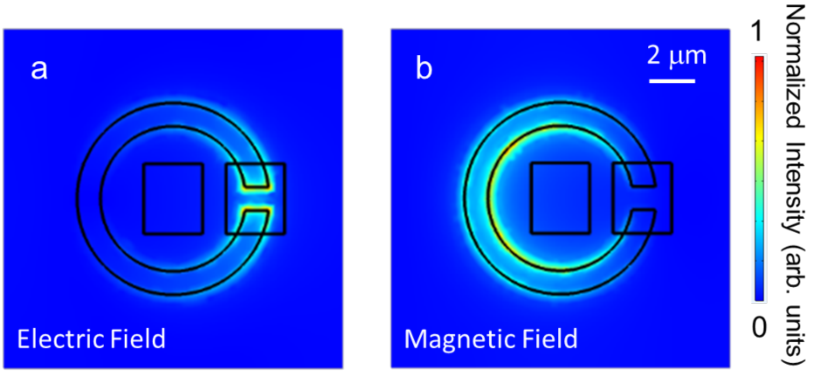

Supplementary figure 12. Electromagnetic Coupling in CSRR. Bi-dimensional normalized intensity of the field distribution of the CSRR at the resonance, for the electric (a) and magnetic (b) field.

The electric field in the split gap, is by-design, the mechanism responsible for the enhancement of the field for THG. The CSRR design permits a resonance with a higher Q-factor. This is shown in Supplementary figure 13, that plots the maps of the electric field distribution in the devised CSRR and in a split-gap ribbon having the same metal width, split gap and with the same resonance frequency of our CSRR. The field enhancement at the resonance in the split gap is visible in both designs, with comparable values (13 in the SRR and 18 in the split ribbon resonator), but the *Q*-factor in the SRR is significantly larger (Q_{SRR}=13, Q_{ribbon}=1.8). Besides, the optimal geometry of the CSRR allows the realization of a denser array, which is more desirable for a more efficient THG process.

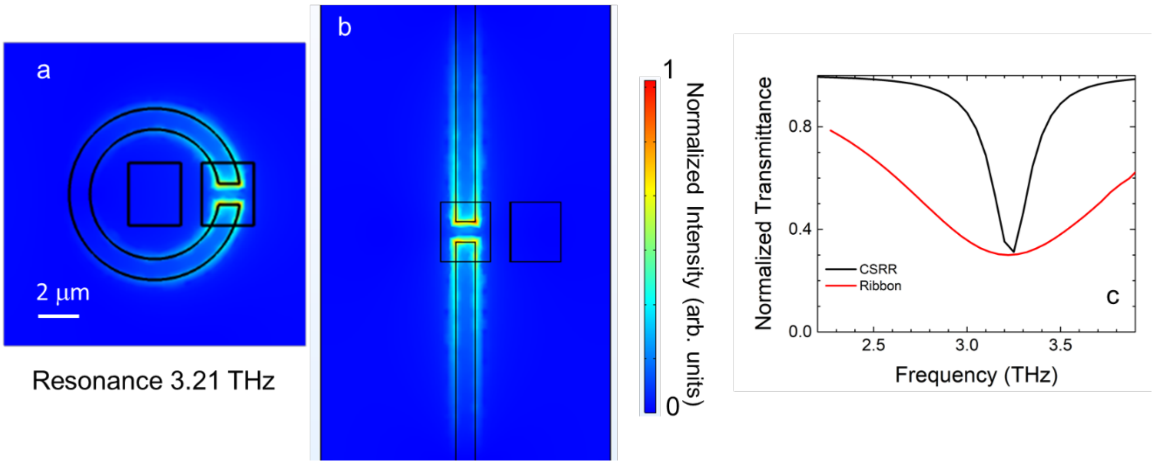

Supplementary figure 13. Resonator Electric Filed Enhancement a,b) Bi-dimensional profile of the normalized electric field intensity at resonance (ν_0 =3.2THz) obtained in the CSRR employed in this work (a), and in an equivalent linear split-gap ribbon (b), whose geometry was designed to have the same split-gap, metal ribbon width and resonant frequency of a). c) Comparison of the calculated transmittance for the CSRR in a) (black curve) and split-gap ribbon (red curve).

Supplementary References

1. Ferrari A.C. *et al.* Raman Spectrum of Graphene and Graphene Layers. *Phys. Rev.Lett* **97**, 187401 (2010).
2. Ferrari A.C.&Basko D.M. Raman spectroscopy as a versatile tool for studying the properties of graphene *Nature Nanotechnology* 8, 235–246 (2013)
3. Calcado L.G. et al. Quantifying Defects in Graphene via Raman Spectroscopy at Different Excitation Energies *Nano Lett.* 11, 3190–3196 (2011).
4. Das, A. *et al.* Monitoring dopants by Raman scattering in an electrochemically top-gated graphene transistor. *Nat. Nanotechnol.* **3**, 210–215 (2008).
5. Robinson, J. A. *et al.* Correlating Raman Spectral Signatures with Carrier Mobility in Epitaxial Graphene: A Guide to Achieving High Mobility on the Wafer Scale. *Nano Lett.* **9**, 2873–2876 (2009).
6. Bruna, M. *et al.* Doping Dependence of the Raman Spectrum of Defected Graphene. *ACS Nano* **8**, 7432–7441 (2014).
7. Mak, K. F., Ju, L., Wang, F. & Heinz, T. F. Optical spectroscopy of graphene: From the far infrared to the ultraviolet. *Solid State Commun.* **152**, 1341–1349 (2012).
8. Frenzel, A. J., Lui, C. H., Shin, Y. C., Kong, J. & Gedik, N. Semiconducting-to-Metallic Photoconductivity Crossover and Temperature-Dependent Drude Weight in Graphene. *Phys. Rev. Lett.* **113**, 56602 (2014).
9. Horng, J. *et al.* Drude conductivity of Dirac fermions in graphene. *Phys. Rev. B* **83**, 165113

(2011).

10. Di Gaspare, A. *et al.* Self-Induced Mode-Locking in Electrically Pumped Far-Infrared Random Lasers. *Adv. Sci.* **10**, 2206824 (2023).

11. Milias, C. *et al.* Metamaterial-Inspired Antennas: A Review of the State of the Art and Future Design Challenges. *IEEE Access* **9**, 89846–89865 (2021).

12. Shahounvand, H., Fard, A. & Tavakoli, M. B. Design, simulation and fabrication of a new terahertz cross-shaped metamaterial bandpass filter to obtain a narrow frequency bandwidth. *Opt. Quantum Electron.* **54**, 120 (2022).

13. Soavi G. et al. Broadband, electrically tunable third-harmonic generation in graphene *Nature Nanotechnology* **13**, 583–588 (2018).

14. Massicotte, M., Soavi, G., Principi, A. & Tielrooij, K.-J. Hot carriers in graphene – fundamentals and applications. *Nanoscale* **13**, 8376–8411 (2021).

15. Tomadin, A., Brida, D., Cerullo, G., Ferrari, A. C. & Polini, M. Nonequilibrium dynamics of photoexcited electrons in graphene: Collinear scattering, Auger processes, and the impact of screening. *Phys. Rev. B* **88**, 35430 (2013).

16. Bonini N., Lazzeri M., Marzari N.,Mauri F., Phonon Anharmonicities in Graphite and Graphene. *Phys. Rev. Lett.* **99**, 176802 (2007).

17. Viljas, J. K. & Heikkilä, T. T. Electron-phonon heat transfer in monolayer and bilayer graphene. *Phys. Rev. B* **81**, 245404 (2010).

18. Lui, C. H., Mak, K. F., Shan, J. & Heinz, T. F. Ultrafast Photoluminescence from Graphene. *Phys. Rev. Lett.* **105**, 127404 (2010).

19. Dawlaty, J. M. *et al.* Measurement of the optical absorption spectra of epitaxial graphene from terahertz to visible. *Appl. Phys. Lett.* **93**, 131905 (2008).

20. Brida, D. *et al.* Ultrafast collinear scattering and carrier multiplication in graphene. *Nat. Commun.* **4**, 1987 (2013).

21. Sun, D. *et al.* Ultrafast Relaxation of Excited Dirac Fermions in Epitaxial Graphene Using Optical Differential Transmission Spectroscopy. *Phys. Rev. Lett.* **101**, 157402 (2008).

22. Han, J. W. *et al.* Plasmonic Terahertz Nonlinearity in Graphene Disks. *Adv. Photonics Res.* **3**, 2100218 (2022).

23. Di Gaspare, A. *et al.* Electrically Tunable Nonlinearity at 3.2 Terahertz in Single-Layer Graphene. *ACS Photonics* (2023) doi:10.1021/acsphotonics.3c00543.

24. Kim, S. *et al.* High-harmonic generation by resonant plasmon field enhancement. *Nature* **453**, 757–760 (2008).

24. Padilla, W. J., Basov, D. N. & Smith, D. R. Negative refractive index metamaterials. *Mater.*

Today **9**, 28–35 (2006).

25. Rahm, M., Li, J.-S. & Padilla, W. J. THz Wave Modulators: A Brief Review on Different Modulation Techniques. *J. Infrared, Millimeter, Terahertz Waves* **34**, 1–27 (2013).
